# Maximizing Small Biopsy Patient Samples: Unified RNA-Seq Platform Assessment of over 120,000 Patient Biopsies

**DOI:** 10.3390/jpm13010024

**Published:** 2022-12-22

**Authors:** P. Sean Walsh, Yangyang Hao, Jie Ding, Jianghan Qu, Jonathan Wilde, Ruochen Jiang, Richard T. Kloos, Jing Huang, Giulia C. Kennedy

**Affiliations:** Veracyte, Inc., 600 Shoreline Court, South San Francisco, CA 94080, USA

**Keywords:** RNA sequencing, lung cancer, interstitial lung disease, thyroid cancer, small biopsy, diagnostics, precision medicine

## Abstract

Despite its wide-ranging benefits, whole-transcriptome or RNA exome profiling is challenging to implement in a clinical diagnostic setting. The Unified Assay is a comprehensive workflow wherein exome-enriched RNA-sequencing (RNA-Seq) assays are performed on clinical samples and analyzed by a series of advanced machine learning-based classifiers. Gene expression signatures and rare and/or novel genomic events, including fusions, mitochondrial variants, and loss of heterozygosity were assessed using RNA-Seq data generated from 120,313 clinical samples across three clinical indications (thyroid cancer, lung cancer, and interstitial lung disease). Since its implementation, the data derived from the Unified Assay have allowed significantly more patients to avoid unnecessary diagnostic surgery and have played an important role in guiding follow-up decisions regarding treatment. Collectively, data from the Unified Assay show the utility of RNA-Seq and RNA expression signatures in the clinical laboratory, and their importance to the future of precision medicine.

## 1. Introduction

Large-scale efforts such as The Cancer Genome Atlas (TCGA) have contributed to the identification of genes and pathways that drive various disease states [1,2]. Many of these efforts have focused on using DNA to elucidate the role of genomic variants in disease progression and treatment [3,4].

The clinical utility of DNA markers in diagnosing disease and selecting treatment is undisputable; however, there are limitations to the information that can be derived from DNA alone. Notably, variants present in DNA may not be expressed in the tissue of interest. Available literature describing disease-associated DNA variants varies widely across disease types, and emerging evidence suggests that tissue-restricted, allele-specific, or imprinted expression of certain genes may modify the influence of genetic variants on disease manifestation [5,6,7,8,9]. In addition, the number of cancers for which the canon of known DNA mutations is sufficient to diagnose disease with reasonably high sensitivity and specificity is limited, leading to underdiagnosis. The use of pan-cancer DNA panels has been a valuable step forward in guiding treatment for patients harboring relevant mutations [10,11]; however, until early diagnosis is improved, many patients will be unable to benefit from subsequent treatment.

The diagnostic shortcoming of DNA variants has led to the widespread development and success of diagnostic algorithms, such as classifiers trained using machine learning on RNA-sequencing (RNA-Seq) derived features [12,13,14,15,16,17]. The potential benefits of applying RNA exome sequencing are wide-ranging. Unlike quantitative reverse transcription PCR (RT-qPCR) and microarrays, which are closed platforms for detecting and quantitating a predefined set of transcripts, use of an open system that interrogates the entire RNA exome through sequencing allows an extensive repertoire of variation to be detected and quantitated without a priori knowledge of the transcripts of interest [18]. Furthermore, in addition to quantifying expression through gene counts, sequencing of RNA transcripts enables the detection and identification of both known and novel translocation (e.g., fusion) events. Single-nucleotide variants (SNVs), and insertions and deletions (indels) that are present in germline or somatic DNA, can also be detected by RNA-Seq if the gene is expressed in a particular specimen (i.e., expressed variants) [18,19,20]. Additionally, the mitochondrial genome can be interrogated by measuring quantitative expression of transcripts, as well as variant detection [21,22].

While whole-transcriptome or RNA exome profiling is often performed in research and discovery efforts, such a complex assay platform is more difficult to implement in a clinical diagnostic setting, owing to robustness, cost and/or throughput issues. Furthermore, addition of a separate DNA assay platform to an RNA workflow to derive variant information adds operational complexity and cost. As a result, the use of targeted, closed platforms often prevails.

**Figure 1:**
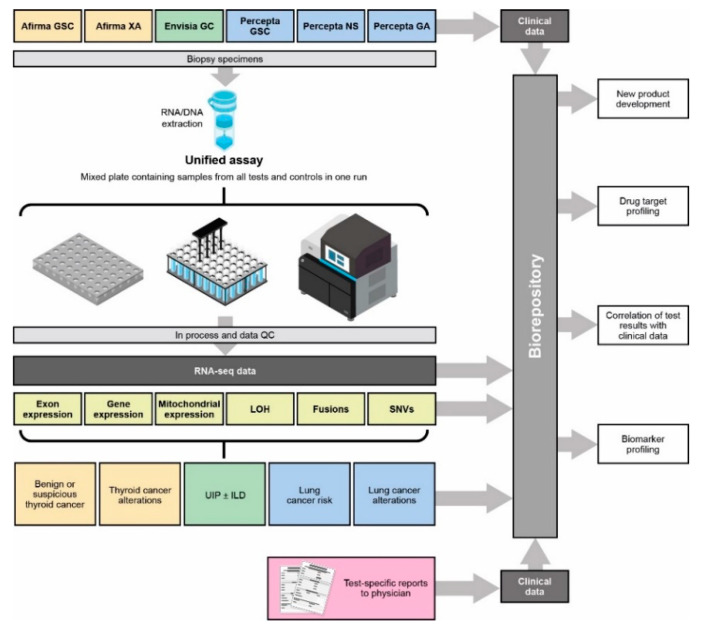
Unified Assay workflow. GA, genomic Atlas; GC, genomic classifier; GSC, Genomic Sequencing Classifier; ILD, interstitial lung disease; LOH, loss of heterozygosity; NS, nasal swab; SNV, single-nucleotide variant; UIP, usual interstitial pneumonia; XA, Expression Atlas.

In establishing the Unified Assay, we have implemented a comprehensive workflow wherein exome-enriched RNA-Seq assays are performed on patient specimens submitted to a Clinical Laboratory Improvement Amendments (CLIA)-certified laboratory for diagnostic testing (Figure 1). Although only the results from clinically validated classifiers, using a subset of all possible features, are delivered to the clinician in a patient report, the underlying RNA exome profile of each sample is stored in a data repository that can be interrogated further in a variety of ways (e.g., for drug target/biomarker profiling and/or development of new classifiers).

Here, we show how the Unified Assay can be successfully deployed as a clinical application, as exemplified in the results from a large repository of small biopsy specimens processed via an automated and clinically validated RNA-Seq assay.

## 2. Materials and Methods

### 2.1. Machine Learning-Based Classifiers

Sequencing data were analyzed via the following advanced machine learning-based classifiers (all developed/licensed by Veracyte, Inc., South San Francisco, CA, USA) that are applied to the diagnosis of thyroid cancer, interstitial lung disease (ILD), and lung cancer, and may be applicable to other disease indications: the Afirma^®^ Genomic Sequencing Classifier (GSC), which uses RNA exome sequencing information derived from a fine-needle aspiration (FNA) biopsy to classify cytologically indeterminate thyroid nodules as Afirma GSC Benign or Suspicious [12,22,23,24,25]; the Afirma Xpression Atlas (XA), which provides genomic alteration information from FNA samples with an Afirma GSC Suspicious result, or cytologically suspicious for malignancy or malignancy nodule [26]; the Envisia^®^ Genomic Classifier (GC), a diagnostic test that uses a 190-gene expression signature to differentiate between usual interstitial pneumonia (UIP) and non-UIP subtypes in lung transbronchial biopsy (TBB) samples from patients with ILD [13]; the Percepta GSC, which uses RNA exome sequencing information from bronchial brushing samples for classification of lung cancer risk among bronchoscopy nondiagnostic samples [16]; the Percepta Nasal Swab (NS) classifier that uses RNA exome sequencing information from nasal (inferior turbinate) samples to classify the risk of lung cancer in patients with previously detected lung nodules [27]; and the Percepta Genomic Atlas (GA) test, which was developed to detect lung cancer-associated fusions and MET exon-skipping events in diagnostic TBB specimens and transbronchial needle aspirates (TBNAs) [28]. Beyond expression signatures, interrogation of RNA-Seq data in these samples revealed specimens with rare and novel genomic events, including gene fusions, mitochondrial variants, and loss of heterozygosity (LOH).

### 2.2. Sample Processing

Freshly collected biopsy samples were placed into sample tubes containing an RNA preservative (RNAprotect; Qiagen, Germantown, MD, USA) ahead of shipping to the laboratory at refrigerated temperatures (2–8 °C), where they were frozen prior to processing. Sample extraction methods were developed and optimized for each biopsy and tissue type, as described previously [12,13,16,24,25]. Briefly, RNA from thyroid nodule FNAs, lung nodule TBNAs, lung nodule TBBs, lung TBBs, and lung tissue were extracted with the AllPrep Micro Kit (Qiagen, Germantown, MD, USA). RNA was extracted from bronchial brushes of upper airway and nasal swabs using Qiazol and the miRNeasy Mini kit (Qiagen, Germantown, MD, USA). RNA was quantitated using the Promega Quantifluor kit (Promega, Madison, WI, USA) and a Tecan fluorometer (Tecan, San Jose, CA, USA). RNA quality was assessed by Bioanalyzer (Agilent, Santa Clara, CA, USA).

### 2.3. RNA-Seq

RNA was sequenced using the Illumina TruSeq RNA Access kit (Illumina, San Diego, CA, USA). The amount of RNA used for library preparation varied by sample type, i.e., 15 ng for thyroid nodule FNAs and lung TBB samples from ILD patients, and 50 ng for all other lung and nasal samples. Library preparation was performed on a custom, automated Hamilton STAR platform (Hamilton, Reno, NV, USA) and sequenced on the NextSeq 500/550 platform (Illumina). The scripts for automated liquid handling were optimized to accurately aspirate and dispense low reagent volumes (as low as 2 μL), thus enabling use of biopsy specimens yielding very low amounts of total RNA (15 ng). All total RNA samples were size fragmented (~200–300 bp) prior to cDNA synthesis and adaptor ligation. Sixteen PCR cycles were used to amplify all cDNA species (derived from all transcripts in the total RNA sample) using primers complementary to the generic adaptor sequences. The cDNA libraries were then enriched for exon content via two rounds of hybridization to probes designed to capture all human exons. Sequencing was performed using the NextSeq 500/550 platform via 2 × 75 bp paired-end protocols, to an average read depth of 25M paired-end reads per sample.

### 2.4. Bioinformatics Pipeline

RNA-Seq data were processed through the bioinformatics pipeline as described previously [12,14,22,29]. Briefly, binary base call (bcl) files were converted to fastq files using Illumina bcl2fastq and aligned to the human genome using STAR aligner [30]. Samples passing prespecified quality metrics were analyzed further. High-throughput sequencing was used to generate gene-level expression data, including mitochondrial chromosome genes, quantified by discrete counts [31], and the read count data normalized using DESeq2 [32] for stabilizing highly variable genes and variation in sequencing depth. SNV were detected using GATK V3.3 HaplotypeCaller, following best practice for RNA-Seq data [33]. Gene fusions were detected using STAR-Fusion [34].

### 2.5. Downstream Analysis

Expression-level data and variant- and fusion-calling results were passed to the Afirma GSC, Afirma XA, Envisia GC, Percepta GSC, Percepta NS, and Percepta GA, and scores and indication-specific predictions were generated by each classifier and biomarker detector. Data processing and visualization were performed with R [35]. Specifically, multiple R packages were used for visualization: ggplot2 [36], networkD3 [37], pheatmap [38], waterfalls [39], circlize [40], and treemapify [41].

### 2.6. Fusion Identification

Fusion calls were annotated by FusionAnnotator (part of the STAR-Fusion tool) and identified as having both splice sites matching a known reference splice site (ONLY_REF_SPLICE) or having one or both splice sites not matching a known reference splice site (INCL_NON_REF_SPLICE). The reference splice sites are reference exon junctions as provided by the reference transcript structure annotation GRCh37_gencode_v19. Fusions were further labeled as being either within chromosomes (intrachromosome) or between chromosomes (interchromosome).

### 2.7. Mitochondrial Gene Expression and Hürthle Classification

Transcripts from all 13 protein-coding mitochondrial genes were captured by the RNA-Seq assay; these were critical input features for the Hürthle Cell Index classifier, an integral component of the Afirma GSC [22].

### 2.8. LOH Score Determination

For each chromosome, the LOH score was calculated as the proportion of variants with variant allele frequency (VAF) < 0.2 or > 0.8 among heterozygous variants, excluding those in human leukocyte antigen (HLA) or immunoglobulin genes. Genome-level LOH scores were calculated similarly using variants across chromosomes 1 to 22.

### 2.9. Copy Number Variation (CNV) Identification

To identify CNVs, we employed jointly regulated blocks (JRBs) methodology, whereby increased and decreased JRB expression identified amplification and deletion events, respectively [42].

## 3. Results

### 3.1. Patient Samples

The Unified Assay was used to generate RNA-Seq data from deidentified clinical samples collected during the development and implementation of the aforementioned tests [12,13,14,16,22,24,25,27,28,29,43,44,45] and processed through the CLIA-certified laboratory. The RNA exome biorepository described herein is comprised of 120,312 clinical samples across 3 major clinical indications, where each clinical sample was processed and sequenced for 26,268 genes (Table 1).

### 3.2. Use of an “Open” Platform and a Large Cohort Identifies Known and Novel Fusions, Including Rare and Unexpected Variant Combinations

**Figure 2:**
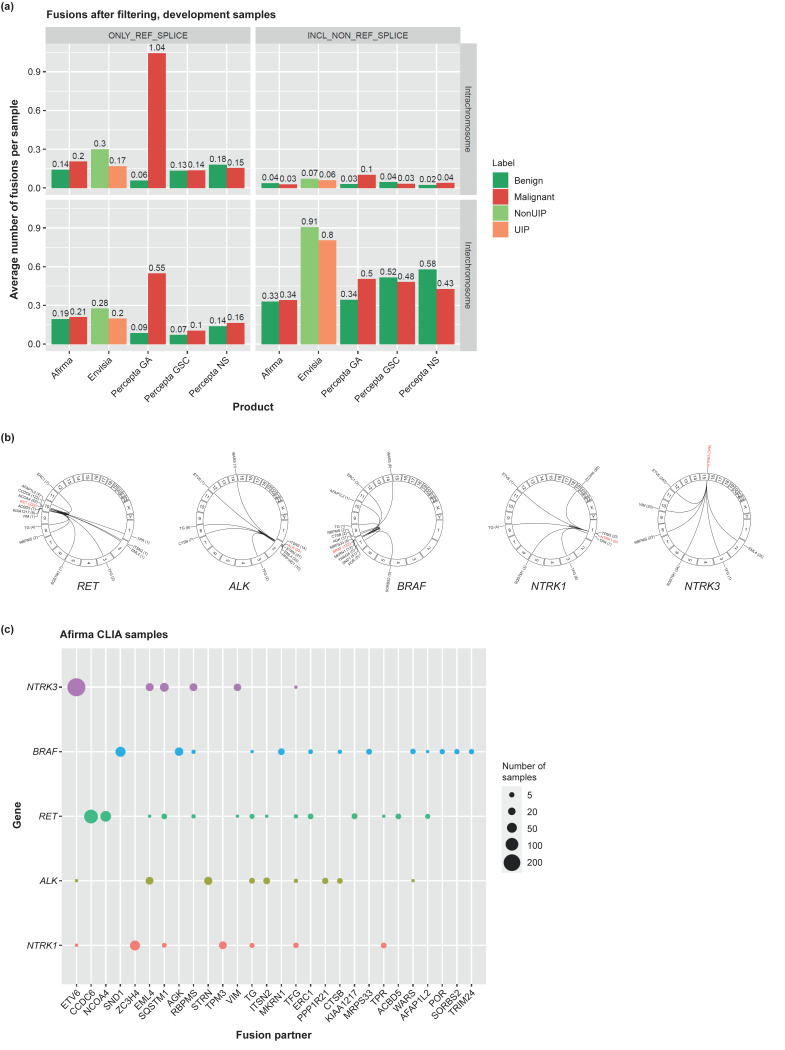
Fusion discovery across Unified Assay samples. (**a**) Number of fusions identified per sample across thyroid (Afirma), ILD (Envisia), and lung (Percepta) samples; (**b**,**c**) canonical fusions identified in thyroid Afirma CLIA samples. The following fusion calls were filtered out: having <3 junction reads; involving HLA, hemoglobin, or mitochondrial genes as one or both fusion partners; being between two immunoglobulin genes; and being called in >10% of samples from any one product with CLIA samples. ONLY_REF_SPLICE = having both splice sites matching a known reference splice site. INCL_NON_REF_SPLICE = having one or both splice sites not matching a known reference splice site. CLIA, Clinical Laboratory Improvement Amendments; GA, Genomic Atlas; GSC, Genomic Sequencing Classifier; HLA, human leukocyte antigen; NS, Nasal Swab; UIP, usual interstitial pneumonia; XA, Expression Atlas.

Use of the Unified Assay enables analysis of the number and types of fusions both across and within chromosomes. Fusions containing known reference splice sites are of particular interest. As shown in Figure 2A, the highest rates of such fusions were observed in malignant lung cancer samples, with a mean of 1.04 intrachromosome and 0.55 interchromosome fusions per sample (Supplementary Figure S1). This rate far exceeded the number of fusions seen in Percepta GA benign samples and in Percepta GSC and Percepta NS samples. Across 159 malignant lung cancer samples, we identified 253 fusions comprising 232 individual fusion pairs, with most individual fusions detected in a single sample. However, several interesting fusions were identified in multiple samples, including *TAOK1-PIPOX* and *LPIN2-MYOM1*, which were each found in 3 samples and have been observed previously in breast and lung cancer TCGA datasets, respectively [50]. Known lung cancer-associated fusions, including *BRAF* and *RET*, were also identified.

ILD is not typically associated with the presence of gene fusions. However, the number of fusions identified per sample within the Envisia ILD set was slightly higher than that observed in the Afirma thyroid set. Accordingly, we explored whether these fusions are relevant to ILD or simply alterations present in normal populations. Within the CLIA patient sample set of 3025 Envisia samples, the most common fusion with a known reference splice site was *TFG-GPR128*, which was also common among Afirma CLIA samples (1.8% across both tests). *TFG-GPR128* has previously been described to have a prevalence of 2% in a healthy population [51], suggesting that this fusion is unlikely to be associated with ILD. Other, rarer fusions were also identified, including *GAS7-MYH1* and *ZCCHC8-RSRC2*, each of which were identified in 4 ILD samples and have been reported previously in cancer samples (*GAS7-MYH1* in lung and stomach adenocarcinoma, and *ZCCHC8-RSRC2* in multiple cancers) [52]. Interestingly, expression of *MYH1* has previously been found to be upregulated in the bleomycin mouse model of idiopathic pulmonary fibrosis (IPF) [53]. Overall, although fusions are rare in ILD, these data indicate the potential for revealing molecular information that may be relevant to disease manifestation.

In total, more than 50,000 fusions (20,527 intrachromosomal and 29,743 interchromosomal fusions with known reference splice sites) containing 36,862 unique fusion pairs were identified in the Afirma CLIA sample set, comprising 109,912 FNAs. Although the number of fusions with known reference splice sites present in thyroid cancer samples was lower than observed for lung cancer samples, it is widely accepted that gene fusions have an important role in thyroid cancer, and drugs have been approved that target some of these (e.g., *RET* and *NTRK*) [54,55]. Evaluation of thyroid FNAs with the Unified Assay identified all fusion proteins described for thyroid cancer in TCGA [50], the most common fusion pair being *PAX8-PPARg*, with other common fusion partners including *RET*, *NTRK1/3*, *ALK*, and *BRAF*. However, while the TCGA study was limited to papillary thyroid carcinoma (PTC) [1], the Unified Assay cohort included all thyroid nodule subtypes (including benign and malignant neoplasia) and was 200× larger than the TCGA study, thus enabling an unbiased analysis of all possible fusion partners for these kinases. Examination of the 109,912 FNAs revealed 353 samples with *NTRK3*, 105 with *NTRK1*, 112 with *ALK*, 274 with *RET*, and 303 with *BRAF* fusions. A subset of fusions with common fusion partners are shown in Figure 2B. *RET* and *BRAF* fusions were the most diverse and included a high level of inter- and intrachromosomal rearrangements, consistent with studies in other cancers [56,57]; however, examination of our larger cohort identified considerably more *BRAF* fusion partners. Along with the observed diversity, no one *BRAF* fusion partner dominated; *SND1/BRAF* and *AGK/BRAF* were the 2 most common fusions. *RET* fusions had the second largest diversity among fusions; however, unlike *BRAF* fusions, *RET* fusions had just 2 frequent partners: *CCDC6* (*RET/PTC1*) and *NCOA4* (*RET/PTC3*). Only 5 *NTRK3* fusion partners were observed in more than two samples, predominated by *ETV6/NTRK3* fusions. Across the different kinase fusions, common partners were observed, with *EML4*, *SQSTM1*, *ERC1*, *ETV6*, and *VIM* all being observed to be fused with multiple kinase partners. Each had a dominant kinase partner, but was also observed with secondary, and sometimes tertiary, fusion partners, indicating that some genes can participate in driver fusions across multiple kinase partners (Figure 2C). Full details of fusions identified using the Unified Assay are provided as supplementary material (Supplementary file S1).

### 3.3. Measuring Mitochondrial Gene Expression Identifies Challenging Subtypes

**Figure 3:**
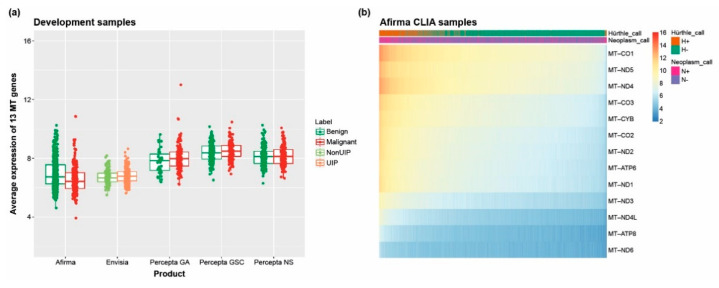
Mitochondrial expression across Unified Assay samples. (**a**) Expression of 13 mitochondrial genes across thyroid (Afirma), ILD (Envisia), and lung (Percepta) samples; (**b**) expression of mitochondrial genes in the subset of thyroid FNAs. Numbers next to the color bar represent the color scale of normalized gene expression, whereby blue indicates low expression values, yellow indicated intermediately expressed genes, and red represents highly expressed genes. CLIA, Clinical Laboratory Improvement Amendments; GA, Genomic Atlas; MT, mitochondrial; UIP, usual interstitial pneumonia.

The capabilities of the Unified Assay extend beyond the measurement of canonical nuclear gene expression, as illustrated by assessment of mitochondrial gene expression across the multitude of patient specimens, revealing both expected and unexpected variations. As shown in Figure 3A, differences in mitochondrial gene expression were observed across different disease states, including lower expression in ILD TBBs compared with lung bronchoscopy brushing and biopsy samples. The application of mitochondrial content for diagnostic purposes is illustrated by the classification of thyroid tumors into the challenging subtype known as Hürthle cell neoplasms, which are characterized by an abundance of malfunctioning mitochondria [58]. We observed significantly higher expression of mitochondrial genes in thyroid FNA samples classified as Hürthle cell-positive based on the Afirma classifier (Figure 3B).

### 3.4. Loss of Heterozygosity Can Be Measured by the Unified Assay

**Figure 4:**
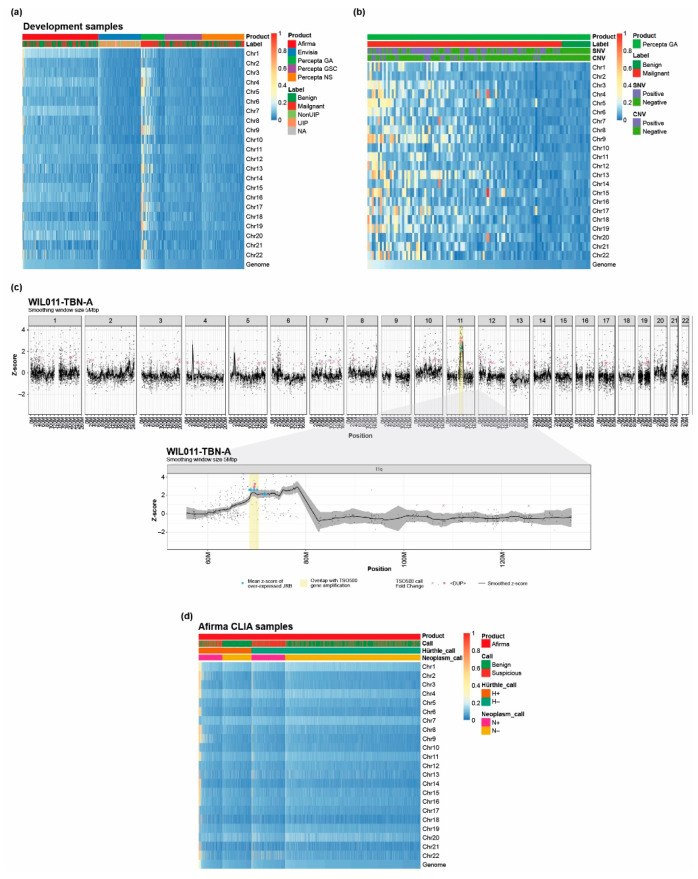
Chromosome and genome-wide LOH across diseases and tissues. (**a**) Chromosome and genome-wide LOH across lung (Percepta), thyroid (Afirma), and ILD (Envisia) samples; (**b**) chromosome and genome-wide LOH across specific lung cancer specimens; (**c**) comparison of results using TSO500 and expression-based CNV identification in an NSCLC-adenocarcinoma sample. The center of the highlighted region on chromosome 11 is gene CCND1, for which a duplication was identified by both TSO500 and expression-based CNV identification; (**d**) chromosome and genome-wide LOH across specific thyroid cancer specimens. Numbers next to the color bar represent the color scale of LOH scores, whereby blue indicates low LOH scores, yellow indicates intermediate LOH scores, and red represents high LOH scores. Expression-based CNV calls were made by the JRB identification method [42], with increased expression JRBs identified as amplification events and decreased expression JRBs as deletion events. Chr, chromosome; CLIA, Clinical Laboratory Improvement Amendments; CNV, copy number variation; GA, Genomic Atlas; GSC, Genomic Sequencing Classifier; ILD, interstitial lung disease; JRB, Jointly Regulated Block; LOH, loss of heterozygosity; NA, not available; NSCLC, non-small cell lung cancer; NS, Nasal Swab; SNV, single-nucleotide variant; TBNA, transbronchial needle aspiration; TSO500, TruSight Oncology 500; UIP, usual interstitial pneumonia.

We exploited the ability to sequence exonic SNVs to identify blocks in expressed transcripts where observed variant allele frequencies were close to 0 or 1, enabling detection of copy number abnormality in the form of LOH. Examination of 194 lung cancer specimens indicated numerous instances of LOH, with the highest LOH scores observed in chromosomes 9 and 13, and the lowest in chromosomes 2 and 20 (Figure 4A,B). Interestingly, at least one lung cancer sample with high LOH could be identified for all 22 chromosomes. A range of LOH scores was observed across malignant lung cancer samples, across alteration (SNV/CNV)-positive/-negative samples, and within and across chromosomes (Figure 4B; see Supplementary Materials [Supplementary file S2] for LOH scores and SNV/CNV calls for these samples). However, in cases where more than one specimen was tested per patient, consistent LOH scores were observed across samples from the same patient. A high degree of LOH was observed across multiple chromosomes in samples without detected CNV or other common alterations, and LOH was detected in chromosomes beyond those identified by a targeted CNV assay. Finally, in addition to LOH scores, the Unified Assay detected specific CNVs within each chromosome (Figure 4C).

We also found LOH in Hürthle classifier-positive thyroid specimens, consistent with previous findings (Figure 4D) [22,59,60]. Examination of Afirma CLIA specimens identified 1063 samples with genomic LOH levels > 0.2, including 973 samples that were Hürthle-classifier positive. High LOH, including near haploidization, has been observed previously in hepatocellular carcinoma [22,59,60], and was also observed in our training set [24]. Patient samples with LOH events on chromosomes 5, 7, 12, and 20 were rare, whereas other chromosomes showed high levels of chromosome-wide LOH across multiple patients.

In contrast to lung and thyroid samples, high LOH was not observed across 359 ILD samples (Figure 4A); the same observation was also true for a larger sample size of ~3000 Envisia ILD samples (data not shown).

### 3.5. Scanning the Transcriptome of Clinical Samples for Drug Targets Reveals Potentially Useful Therapeutic Information

**Figure 5:**
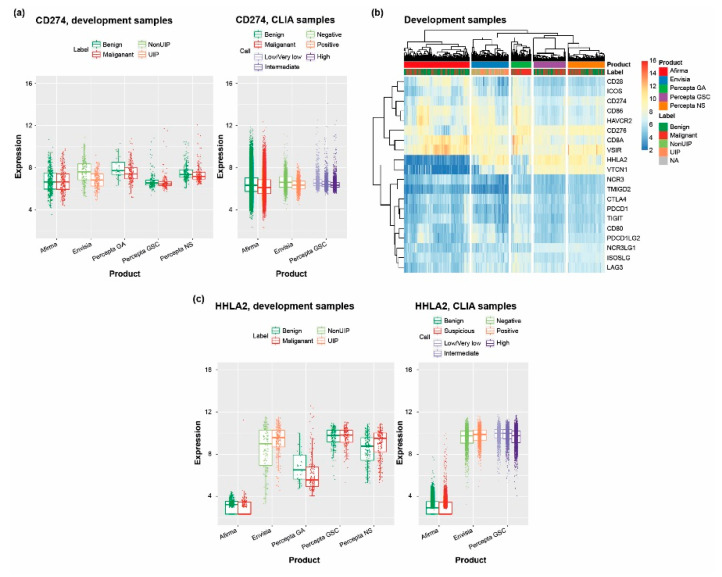
RNA and gene expression across samples used in the development of each test. (**a**) RNA expression of CD274 (PD-L1); (**b**) gene expression of potential drug targets (focusing on B7 ligands and receptors), whereby blue indicates low expression values, yellow indicated intermediately expressed genes, and red represents highly expressed genes; (**c**) RNA expression of HHLA2. CLIA, Clinical Laboratory Improvement Amendments; GA, Genomic Atlas; GSC, Genomic Sequencing Classifier; HHLA2, HERV–H LTR-associating protein 2; NA, not applicable; NS, Nasal Swab; UIP, usual interstitial pneumonia; PD-L1, programmed death-ligand 1.

The immune response to cancer and other diseases is a key area of interest in both therapeutic and biomarker development. Therefore, we used the Unified Assay to investigate the expression of programmed death-ligand 1 (PD-L1) and other related genes. PD-L1 (CD274) was expressed in both benign and malignant thyroid and lung cancer samples, as well as within the nasal epithelium (Percepta NS), upper airway (Percepta GSC), and ILD samples (Envisia), with higher expression in benign versus malignant lung cancer samples and in non-UIP versus UIP ILD samples (Figure 5A). Expression of genes encoding other B7 family ligands and receptors, and additional key components of this signaling system, also varied across the development sample sets for each assay (Figure 5B). Expression of genes encoding two B7 family ligands, *B7-H4* (*VTCN1*) and *B7-H7* (*HHLA2*), showed a large degree of variation across different disease states. Expression of *HHLA2* was lower in thyroid cancer and lung cancer samples compared with other respiratory-associated samples, and higher in UIP versus non-UIP ILD samples (Figure 5C).

## 4. Discussion

### 4.1. Establishment of an Innovative Platform for Clinical Diagnostic Testing

The Unified Assay described herein provides a wealth of data that can be used to support accurate diagnoses and/or identify risk factors in challenging diseases, guide treatment decisions, and have the potential to enable further discoveries that can unlock future advances in patient care and the management of various diseases.

Although RNA-Seq has enormous clinical potential, this technique has yet to be widely deployed in clinical laboratories due to both assay and analysis complexity, including the variable nature of sample quality, assay reproducibility, and robustness of the analysis pipeline [18,61]. Notwithstanding these challenges, the data presented herein show the powerful utility of RNA-Seq in the clinical diagnostic laboratory.

Several clinical tests across different diseases and biopsy types have been run on the Unified Assay platform, with each test having been analytically and clinically validated, and shown to be robust to normal laboratory variation [12,13,16,24,25,44]. In addition, the data can be examined in a variety of ways. A summary of these features and other key properties of the Unified Assay platform is provided in Table 2.

### 4.2. Use of an Enrichment-Based Approach to Facilitate Diagnosis and Treatment Decisions

The Unified Assay uses an enrichment-based approach whereby, following cDNA amplification, the resulting PCR products are hybridized to a cocktail of oligonucleotide probes designed to enrich for all exons of 26,268 genes. Further customization can be achieved by tiling extra enrichment probes over key exons and adding probes in non-coding regions (e.g., untranslated regions, introns, intergenic regions). Compared with total or nonenriched RNA-Seq, this exon-enrichment approach leads to approximately 5-fold lower sequencing costs and the ability to obtain significantly greater read depth for the key coding regions (exons), while retaining the ability to measure a wide range of genomic features (e.g., SNVs, fusions, mitochondrial gene expression, LOH), all from a single test, on a preciously small biopsy sample. Unlike amplicon-based RNA-Seq, which requires locus-specific PCR primer design [62], this enrichment approach can detect novel genomic events such as fusion partners not previously known to exist, and not anticipated by any bespoke assay design.

The breadth and richness of the data generated by the Unified Assay is exemplified by the Afirma GSC/XA, which uses multiple data features to facilitate diagnosis and treatment decisions for suspected thyroid cancer. For example, detection and quantitation of both nuclear and mitochondrial transcripts increases the information available for diagnostic purposes. Chronic lymphocytic thyroiditis (benign), Hürthle cell adenomas (benign), and Hürthle cell carcinomas (malignant) are observed among thyroid nodules and are characterized by increased abundance of mitochondria relative to non-Hürthle nodules [58]. Differentiating which samples require surgery has been a long-standing clinical challenge. Instead of relying on cytopathology or histopathology diagnoses, which are highly variable (and the latter of which requires surgical resection [63]), we used the presence of mitochondrial genes to develop a Hürthle-specific classifier that uses RNA-Seq expression and mitochondrial transcript data to routinely identify FNAs containing Hürthle cell features. These FNAs are then interrogated by an additional classifier that utilizes RNA-Seq expression and chromosomal level LOH to differentiate neoplastic from non-neoplastic samples [22]. Partnering this process with a 12-classifier expression-based “Ensemble Model”, the Afirma GSC renders either a highly accurate result of “Benign”, which facilitates clinical observation in lieu of diagnostic surgery, or renders a result of “Suspicious”, where the yield of malignancy is significantly increased.

The Unified Assay has also been deployed for other diagnostic tests, including a classifier that can utilize small biopsies to distinguish the UIP pattern associated with IPF from the non-UIP patterns seen in other ILDs [13,29]. Analysis of whole-transcriptome data from Envisia CLIA samples also enables investigation of other potential biomarkers that may be relevant to ILD diagnosis. While we found no evidence of changes in mitochondrial gene expression or LOH signal across the ILD samples, we did identify several non-canonical fusions whose association with ILD behavior could be explored further. In addition, ILD was found to share some similarities with lung cancer, such as variation in genes involved in cell proliferation and immune signaling, which is notable given the existence of shared risk factors for lung cancer and ILD (e.g., smoking, older age, male sex) [64]. Current lung cancer biomarker testing focuses on specific variants and fusions that are associated with existing therapeutics; however, RNA-Seq data can be used to add to and extend these investigations. Identification of novel fusion partners may help to reveal more patients with fusions involving currently targetable kinases such as *RET* and *ALK*, and, although the biological significance of many such fusions is yet to be determined, they could potentially become the therapeutic targets of tomorrow.

### 4.3. Identification of Disease-Related Variants by Comprehensive Testing

Robust functional testing of reagents and the use of pre-tested control reagents alongside maintained and validated equipment, within a full suite of standard operating procedures, minimizes technical batch effects and enables the combination and analysis of data sets that were run months, and even years, apart and most importantly, across both development and CLIA samples. Additionally, use of a whole-transcriptome assay yields copious data that can be used for quality control analysis to reduce technical noise and improve detection of biological signals. Quality-control methods include identifying gender through expressed genes and use of expressed single-nucleotide polymorphisms [12], not only to identify potential technical contamination events, but also to link samples from the same patient and confirm accuracy of associated clinical data, which is particularly useful in longitudinal studies.

Eliminating the need for bridging to targeted assays means all important features from development to commercial use could be preserved. For example, there is no limit to the number of genes that can be used in the Unified Assay, whereas targeted assays are limited, e.g., to tens to hundreds of genes. Each targeted assay type is also different and, when bridging to a new platform, not all genes or features will perform equivalently, thus necessitating further rounds of optimization.

Most importantly, however, targeted assays may not be able to generate data on all feature types. By contrast, the use of RNA-Seq enables detection of expressed, and therefore clinically relevant, variants and fusions that are more likely to be important to the etiology of the disease compared with non-expressed variants [8,12,65,66,67]. Allele-specific expression, or allelic imbalance, has been observed in tumors [8,9,12,65,67,68], where it gives rise to expressed RNA allele frequencies that are different from DNA-based allele frequencies. These include both variant-enriched expression (RNA VAF > DNA VAF) and wild-type–enriched expression (RNA VAF < DNA VAF). Indeed, we previously described detection of dramatically different VAFs when comparing DNA and RNA methods for some FNA samples in which a DNA variant was identified, but no variant was identified by whole-transcriptome RNA-Seq [12]. In these cases, the wild-type allele was predominantly expressed, accounting for the lack of variant detection using RNA methods. Treatment selections are often based on the interpretation of a DNA-based assay; however, if the variant is not expressed, then the treatment may not be efficacious [66]. Conversely, when the presence of a variant allele is contraindicative for therapy (e.g., *KRAS* variant positivity and anti-EGFR treatment in colorectal cancer [69]), variant-enriched expression may negatively impact treatment outcomes. Finally, the importance of expressed variants has been recognized in the identification of genetic disorders that may be caused by splicing alterations [6,70]. Such variants classified as variants of unknown significance based on DNA-seq may be resolved using RNA-Seq in some cases, thereby improving the diagnostic yield. Taken together, these observations suggest that RNA-Seq can play an important role in the identification of critical disease-related variants.

### 4.4. RNA-Seq–Based Identification of Potential Therapeutic Targets

Use of the Unified Assay provides new insights into both novel and rare fusions. Analysis of more than 100,000 FNA samples, including both benign and malignant thyroid nodules, identified 895 instances of *NTRK*, *RET*, *ALK* and *BRAF* fusions, representing potentially actionable kinase fusions. Interestingly, rare fusions included some involving *RET* and *NTRK3* that had only been described previously in single-case studies of patients exposed to radiation from the Fukushima nuclear disaster (*SQSTM1/NTRK3*; *AFAP1L2/RET*) [71] or atomic bomb fallout (*ACBD5/RET*) [72], and in a single case of salivary gland carcinoma (*VIM/RET*) [73].

Beyond detecting somatic variants that are potential oncogenic drivers, chromosome and genome-wide variants can be used to analyze LOH and CNV in tumors [22]. While less dense than LOH measurements using genomic DNA, genome-wide LOH using expressed genes is nonetheless a powerful tool for assessing genomic instability. Moreover, this measurement is obtained using the same small quantity of RNA that is used to generate count, mitochondrial, and fusion data, and therefore does not require extraction of a separate DNA/RNA sample. We noted potentially relevant patterns of LOH both across and within tumor (lung and thyroid) types. As well as detecting the same CCND1 CNV that was identified in lung cancer samples using a CNV-specific targeted assay, use of the Unified Assay revealed LOH in multiple chromosomes, thus providing a much more complete picture of the changes within these tumor samples.

Measurements of PD-L1 levels are currently used to identify patients most likely to benefit from certain immune checkpoint inhibitors [74], and the observed variable expression of genes encoding other B7 family members and inhibitory receptors in our patient samples highlights their potential as additional biomarkers and/or therapeutic targets. In this regard, HHLA2 (along with other B7 family members) has been proposed as a potential immunotherapy target in lung cancers [75]. Furthermore, while currently available immune checkpoint inhibitors are known to have immune-related side effects, including development of ILD [76], our findings of upregulation of HHLA2 expression in UIP ILD samples could indicate a potential therapeutic target of interest for IPF. With ongoing advances in the development of new immunotherapies, the need to understand the expression and function of such molecular markers in various diseases and tissues is paramount. Expression of genes encoding B7 family members B7-H1 (PD-L1, CD274), B7-DC (PD-L2, CD274, PDCD1LG2), B7-H2 (ICOSLG), and B7-H3 (CD276) in the airway and nasal epithelium has been demonstrated previously [77], which, our findings indicate, can be extended to other B7 costimulatory molecules B7-H4 (VTCN1), B7-H5 (VSIR), and B7-H6 (HHLA2). Moreover, while HHLA2 has previously been identified as being upregulated in IPF compared with normal lung tissues [78], we also show increased expression of HHLA2 within UIP versus non-UIP ILD subtypes. Notably, several other groups have proposed a role for B7 family members in inflammatory disorders, including allergic rhinitis, asthma, and autoimmune diseases such as arthritis [77,79,80], and a possible role for immune checkpoint inhibitors has been considered in IPF [81]. Therefore, our finding of variable expression of genes encoding additional B7 family members between non-UIP and UIP subtypes suggests further investigation into their role in ILD is warranted.

### 4.5. Realizing the Potential of the Unified Assay

No individual molecular feature described above is sufficient to describe the complexity of disease biology. For example, in the lung cancer data set, LOH scores were different between pathological variants and between CNV-positive and -negative samples. However, combining data on novel expression signatures with expressed fusion, variant, and LOH data may facilitate development of improved biomarker-based diagnostics without the need for additional biopsies or assays. Realizing this potential will require extensive analysis of these large data sets and demonstration of robust and reproducible performance at all stages of the pipeline.

Several key challenges have been identified that contribute to “analysis paralysis” in the development and clinical implementation of RNA-Seq platforms. These include the involvement of complex pipelines encompassing multiple tools that are independently developed, maintained, and licensed, as well as an overabundance of software tools and other options [18]. To address such challenges, carefully designed experiments were conducted to leverage samples with clear biological truth on the genomic alterations under investigation, including technical replicates at each experimental stage. This allowed us to further refine and optimize the pipeline to fit our needs and implement additional, novel normalization techniques to enhance the signal-to-noise ratio. It also enabled us to clearly quantitate all key performance metrics. We have leveraged modern tools such as Docker to modularize each key component to ensure that it is agile and robust, and carefully followed best practice for software development, with comprehensive documentation and thorough validation of each step of the pipeline, as well as the entity of the system. Our pipeline is also deployed on-premises and in-cloud to maximize computational efficiency.

An additional challenge associated with the implementation of RNA-Seq platforms is the lack of consensus by regulatory authorities regarding best practices and reference standards for validation [18]. Although RNA-Seq is now established as a research tool in many laboratories and institutions, these assays are not necessarily standardized, or their reproducibility assessed sufficiently to meet the requirements for a clinical assay. Often, the main aim is to control technical variability within a study to support the answering of specific biological questions within that study. The Unified Assay comprises multiple molecular tests, which were developed using FDA design control guidance [82] and run within the Veracyte CLIA-certified laboratory. Afirma GSC, Envisia, and Percepta GSC all have CLIA and New York State Department of Health approval. To demonstrate the technical and clinical performance of the launched assays, various analytical validation, clinical validation, and clinical utility studies have been performed and published in peer-reviewed journals [12,13,16,24]. Controls for each assay, documented and version-controlled procedures, and demonstration of training and competence of staff have also been implemented as part of CLIA requirements. Demonstration of clinical validity is not required by CLIA regulations but is required as part of the FDA’s in vitro device approval process and, along with clinical utility data, is key to obtaining reimbursement.

## 5. Conclusions

The Unified Assay platform described herein shows the realization of the promise of RNA-Seq in the clinic. Use of RNA-Seq data from thyroid nodules has enabled a complex, multifactorial analysis to examine the molecular nature of individual clinical samples. Notably, of the 109,912 Afirma GSC samples, 75,428 patients received a molecular benign diagnosis, potentially allowing for the prevention of unnecessary surgery and, among the 34,484 patients with a molecular suspicious or high-risk cytopathology result, the use of RNA-Seq helped to identify several important driver events that may inform treatment decisions, including *BRAF* mutations, and fusions in *RET* and *NTRK*. The diagnosis of ILD and lung cancer in thousands of patients has similarly benefitted from the development of classifiers based on robust expression data.

Continued application of multi-modal data that can be accessed from RNA-Seq will help to maximize the information that can be obtained from individual patient biopsies and will ultimately support our scientific understanding of disease biology to expedite the development of new diagnostics and medicines. Indeed, the vast size of this biorepository and the capability of finding associated rare alterations in a large cohort has proven useful to identify patients who may benefit from targeted treatments, in the development of new tests, and in providing enhanced visualization and knowledge to patients, clinicians, and therapeutic partners. Ultimately, we endeavor to enable the biorepository as a platform for interactive research collaboration that will allow for the exploration of additional biological characteristics or genomic signatures beyond the fully developed and validated classifiers. Collectively, these assays show the power and utility of RNA-Seq and RNA expression signatures in the clinical laboratory and their importance to the future of precision medicine.

## Figures and Tables

**Table 1:** Clinical samples [27,45,46,47,48,49].

Indication	Development Samples, n	Classifier (CLIA) Samples, n
Thyroid (Afirma GSC)	634	109,912
ILD (Envisia GC)	359	3025
Lung (Percepta GSC)	311	5521
Lung (Percepta NS)	356	
Lung (Percepta GA)	194	

Development samples are those used in Research and Development to generate RNA-Seq data to train and develop the classifiers. CLIA samples are patient samples collected and tested once a test is commercially available. CLIA, Clinical Laboratory Improvement Amendments; FNA, fine-needle aspiration; GA, Genomic Atlas; GC, Genomic Classifier; GSC, Genomic Sequencing Classifier; ILD, interstitial lung disease; NS, nasal swab; TBB, transbronchial biopsy.

**Table 2:** Key properties of the Unified Assay platform.

Properties	Benefits over Current Diagnostic Tools
Open platform	No a priori knowledge about transcripts needed
Enrichment-based approach	Greater read depth for the key coding regions Ability to measure a wide range of genomic features beyond nuclear gene expression
Wider dynamic range than microarrays and RT-qPCR for detecting expression differences	Detection and semi-quantitative measurement of known and novel variants in transcribed genes Improved detection of biologically meaningful genomic variants More accurate identification of patients most likely to benefit from targeted therapies
Use of the same analy tically validated clinical assay for both research and CLIA samples	Eliminates the need for bridging of methods to targeted assays Availability of data for quality control analysis Application to all phases of product development Lower cost and faster turnaround time
Detection and quantitation of both nuclear and mitochondrial transcripts	Increased diagnostic information More accurate identification of patients most likely to benefit from targeted therapies
Creation of complex RNA signatures for diagnosis as well as providing prognostic and/or predictive information using all transcripts as features in machine learning	Potential for more accurate diagnosis Means to avoid unnecessary diagnostic surgery
Detection and quantitation of known and novel translocations/fusions	Discovery of fusions that may be critical to tumorigenesis/disease etiology Identification of potential therapeutic targets to slow or halt disease progression
Chromosome and genome-level LOH measurements, and identification of specific CNVs	Can assess genomic instability May facilitate development of novel anticancer drugs
Collection of full transcriptome data on every patient sample, creating a large biorepository for Pharma to mine	Maximizes the data accessible from a small biopsy Integration with clinical data facilitates identification of patients who may benefit from targeted treatments Identification of rare events may reveal new drug targets Allows large-scale biomarker profiling

CLIA, Clinical Laboratory Improvement Amendments; CNV, copy number variation; LOH, loss of heterozygosity; RT-qPCR, real-time quantitative polymerase chain reaction.

## Data Availability

The raw (>100,000 exome level sequencing file) datasets generated and/or analyzed during the study are impractical to archive; however, the authors confirm that the data supporting the findings of this study are available within the article and its supplementary materials.

## Supplementary Materials

The following supporting information can be downloaded at: https://www.mdpi.com/article/10.3390/jpm13010024/s1, Figure S1: Number of fusions identified per sample across benign and malignant lung samples; Supplementary file S1: All filtered fusions; Supplementary file S2: Percepta GA_LOH.
