# Supplementary figures and images for: Maximizing Small Biopsy Patient Samples: Unified RNA-Seq Platform Assessment of over 120,000 Patient Biopsies
Source: J Pers Med. 2022 Dec 22;13(1):24. doi: 10.3390/jpm13010024 (PMC9866839; doi:10.3390/jpm13010024)

**Percepta GA, ONLY\_REF\_SPLICE**

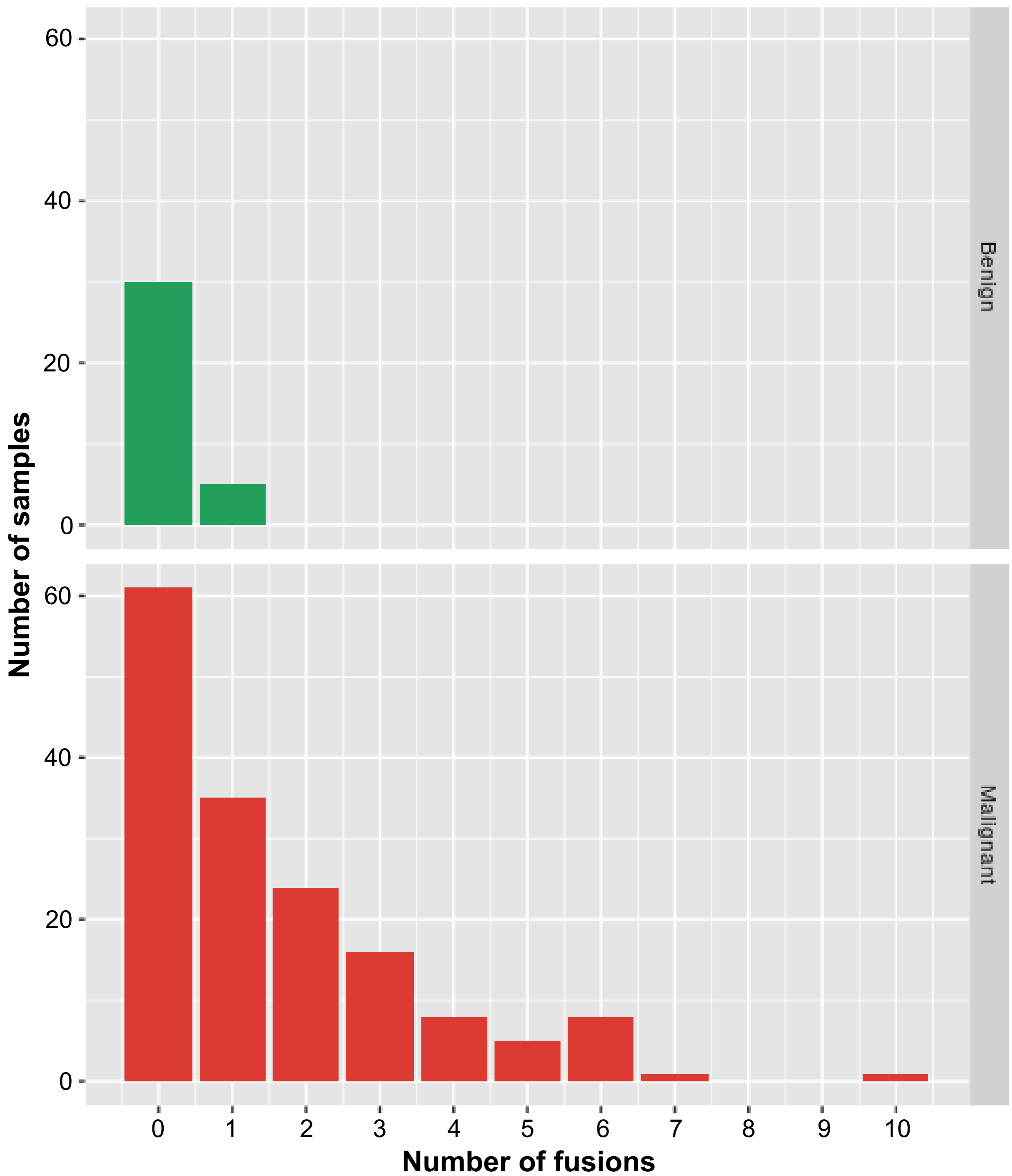

Supplement: Supplementary file 1 [file jpm-13-00024-s001.zip › Supplemental Figure S1.pdf]
